# Supplementary material for: KCNQ1 p.D446E Variant as a Risk Allele for Arrhythmogenic Phenotypes: Electrophysiological Characterization Reveals a Complex Phenotype Affecting the Slow Delayed Rectifier Potassium Current (IKs) Voltage Dependence by Causing a Hyperpolarizing Shift and a Lack of Response to Protein Kinase A Activation
Source: Int J Mol Sci. 2024 Jan 12;25(2):953. doi: 10.3390/ijms25020953 (PMC10816087; doi:10.3390/ijms25020953)
Supplement: Supplementary file 1 [file ijms-25-00953-s001.zip › ijms-2769364-supplementary.pdf]

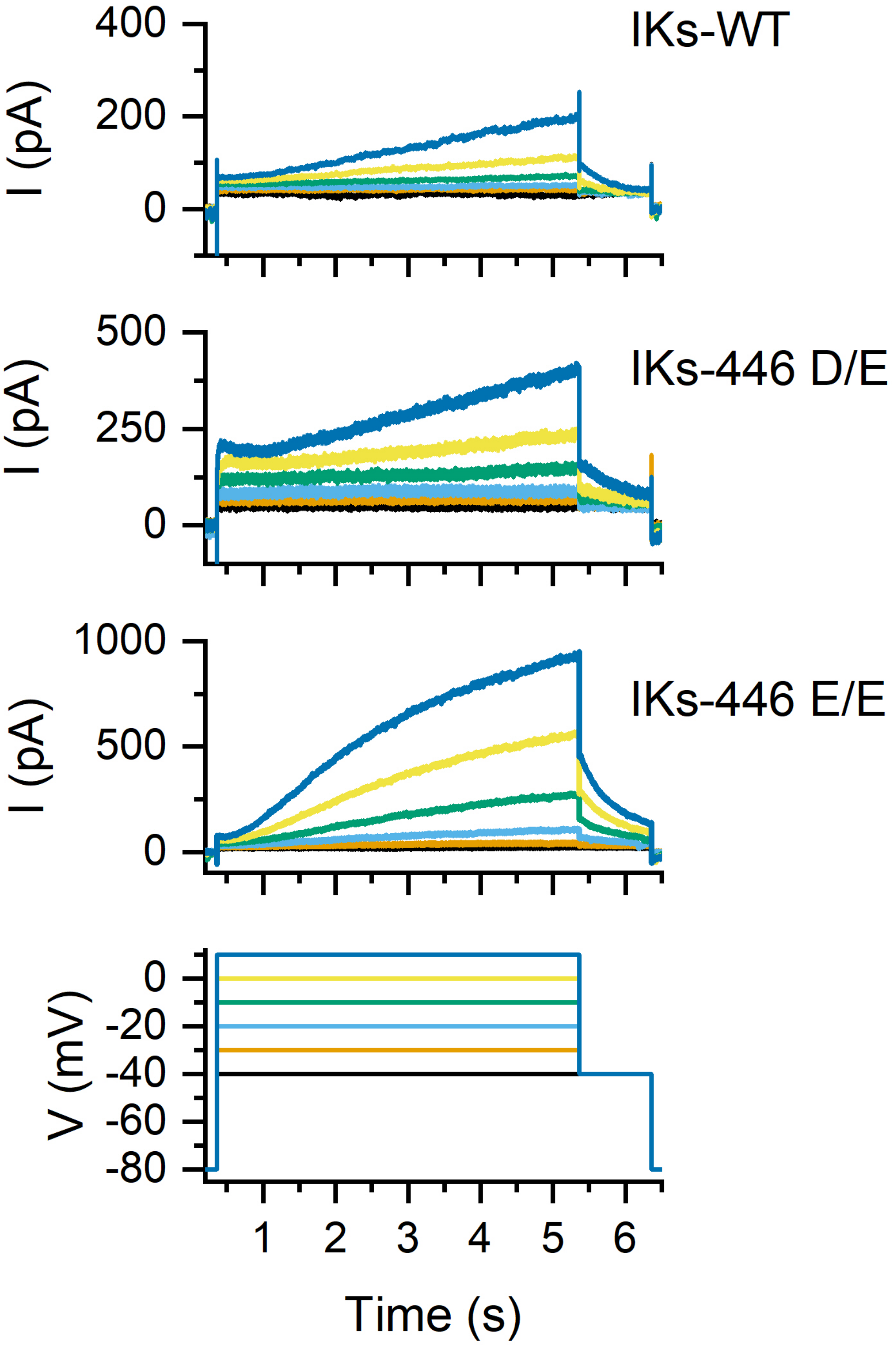

Figure S1. Representative traces of experimental series without 8Br-cAMP at more negative potentials.

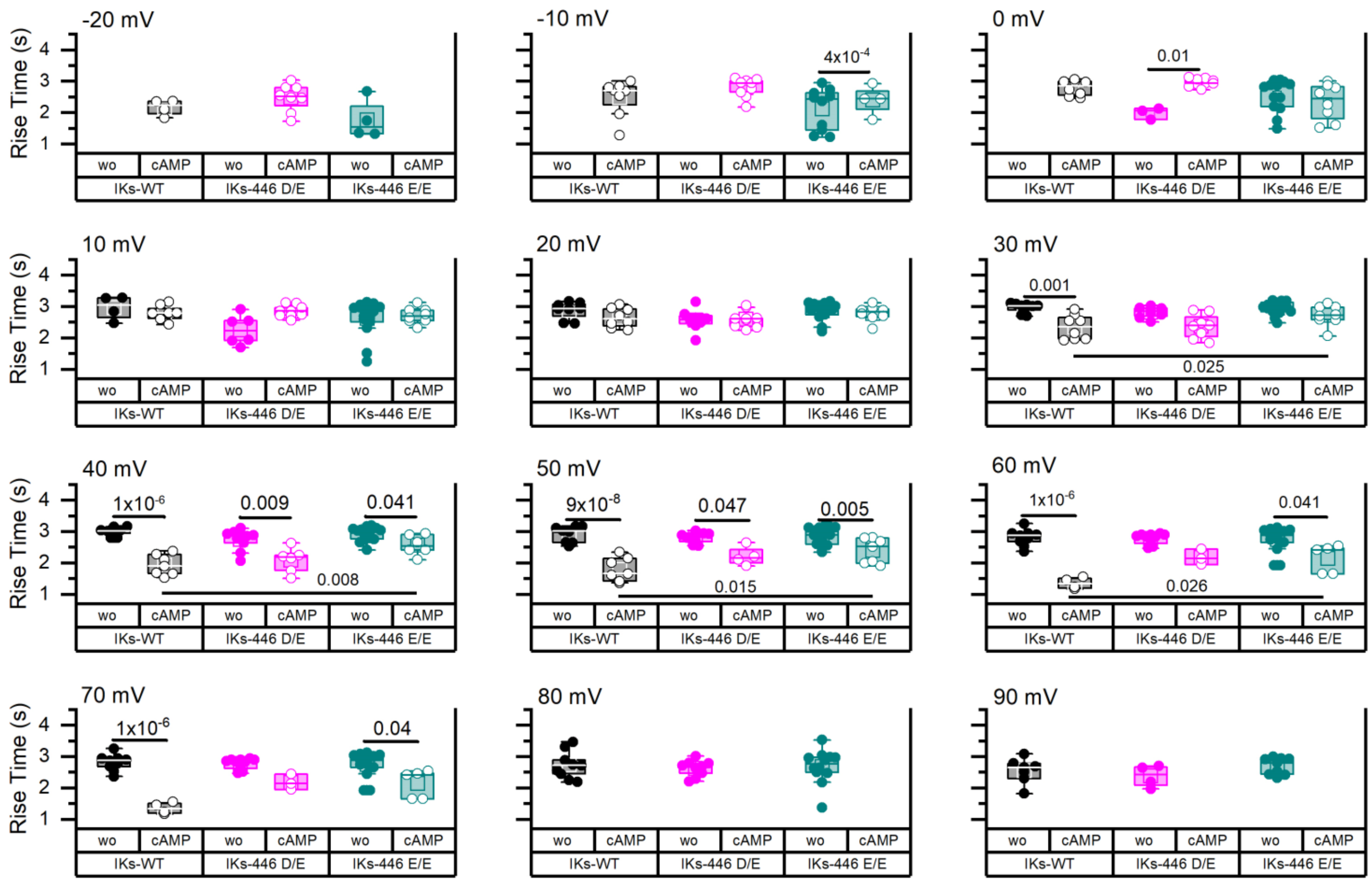

Figure S2. Box plots of rise time values at -20 to 90mV. Data are presented as means ± SEM. The *p* values of statistically significant differences as assessed by two-way ANOVA and Tukey´s comparison test are indicated.

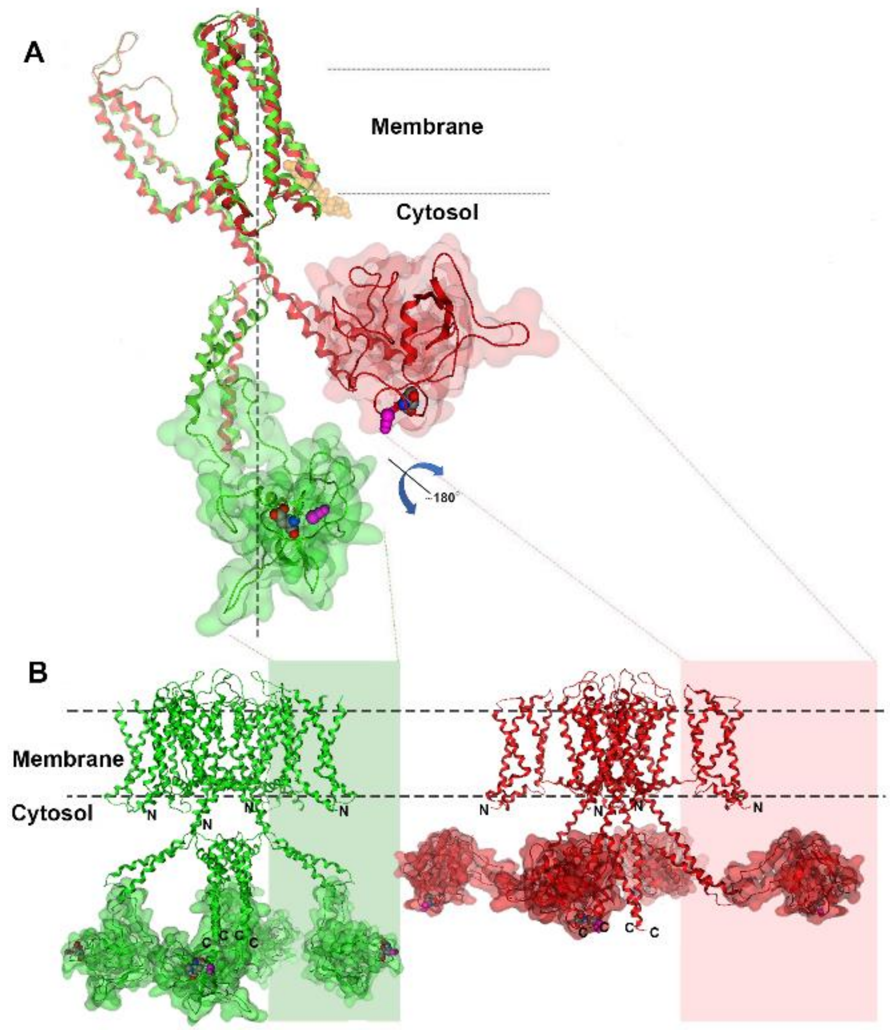

Figure S3. Structural model of the Kv7.1 channel. The calmodulin molecule was intentionally excluded from Figure 4 to emphasize the channel chain alterations. As in Figure 4, the attached conformation is depicted in green, while the detached conformation is presented in red. The amino and carboxyl domains are denoted by N and C, respectively. **A,** Superposition of Kv7.1 monomers highlighting the dynamic movement of our modeled domain 5 upon detachment from the VSD domain. Domain 5 is delineated by green or red outlines. **B,** Kv7.1 tetramers in attached (left) and detached (right) conformations, with the same color codes as in panel A except for calmodulin, which is depicted in yellow in both cases. The sizes of the amino- and carboxyl- domains are comparable to that of domain 5. N and C represent the locations of their respective chains within the cytosol.

Table S1. *P* values for rise time comparisons among genotypes for a 10 to 70 mV range, using Tukey´s comparison test from two-way ANOVA.

10 mV

Genotype	8Br-cAMP	Genotype	8Br-cAMP	MeanDiff	SEM	q Value	Prob	Alpha	Sig*	LCL	UCL
446 D/D (WT)	8Br-cAMP	446 D/D (WT)	wo	-180.92628	246.18711	1.03932	0.97644	0.05	0	-914.30065	552.44808
446 E/E	wo	446 D/D (WT)	wo	-335.6572	226.23034	2.09826	0.67615	0.05	0	-1009.58173	338.26733
446 E/E	wo	446 D/D (WT)	8Br-cAMP	-154.73092	176.00418	1.24328	0.94954	0.05	0	-679.03518	369.57334
446 E/E	8Br-cAMP	446 D/D (WT)	wo	-251.0252	246.18711	1.44201	0.90885	0.05	0	-984.39957	482.34916
446 E/E	8Br-cAMP	446 D/D (WT)	8Br-cAMP	-70.09892	201.01094	0.49318	0.99927	0.05	0	-668.89658	528.69874
446 E/E	8Br-cAMP	446 E/E	wo	84.632	176.00418	0.68003	0.99661	0.05	0	-439.67226	608.93626
446 D/E	wo	446 D/D (WT)	wo	-709.58702	259.504	3.86702	0.08853	0.05	0	-1482.63148	63.45744
446 D/E	wo	446 D/D (WT)	8Br-cAMP	-528.66074	217.11662	3.44349	0.16673	0.05	0	-1175.43614	118.11466
446 D/E	wo	446 E/E	wo	-373.92982	194.19501	2.72312	0.40082	0.05	0	-952.42332	204.56368
446 D/E	wo	446 E/E	8Br-cAMP	-458.56182	217.11662	2.98689	0.30012	0.05	0	-1105.33721	188.21358
446 D/E	8Br-cAMP	446 D/D (WT)	wo	-114.52226	241.58508	0.6704	0.99683	0.05	0	-834.18748	605.14297
446 D/E	8Br-cAMP	446 D/D (WT)	8Br-cAMP	66.40403	195.34752	0.48073	0.99936	0.05	0	-515.5227	648.33075
446 D/E	8Br-cAMP	446 E/E	wo	221.13494	169.5073	1.84495	0.78094	0.05	0	-283.81558	726.08547
446 D/E	8Br-cAMP	446 E/E	8Br-cAMP	136.50295	195.34752	0.98821	0.98112	0.05	0	-445.42378	718.42967
446 D/E	8Br-cAMP	446 D/E	wo	595.06477	211.88413	3.97174	0.07483	0.05	0	-36.12339	1226.25292

20 mV

Genotype	8Br-cAMP	Genotype	8Br-cAMP	MeanDiff	SEM	q Value	Prob	Alpha	Sig*	LCL	UCL
446 D/D (WT)	8Br-cAMP	446 D/D (WT)	wo	-235.32325	170.31769	1.95398	0.73759	0.05	0	-739.59851	268.95201
446 E/E	wo	446 D/D (WT)	wo	-14.62301	149.12933	0.13867	1	0.05	0	-456.16392	426.9179
446 E/E	wo	446 D/D (WT)	8Br-cAMP	220.70024	149.12933	2.09293	0.6783	0.05	0	-220.84067	662.24115
446 E/E	8Br-cAMP	446 D/D (WT)	wo	-97.65732	170.31769	0.81089	0.99235	0.05	0	-601.93258	406.61793
446 E/E	8Br-cAMP	446 D/D (WT)	8Br-cAMP	137.66592	170.31769	1.14309	0.96471	0.05	0	-366.60933	641.94118
446 E/E	8Br-cAMP	446 E/E	wo	-83.03432	149.12933	0.78743	0.99332	0.05	0	-524.57523	358.50659
446 D/E	wo	446 D/D (WT)	wo	-469.50117	165.51905	4.01147	0.06761	0.05	0	-959.56863	20.56629
446 D/E	wo	446 D/D (WT)	8Br-cAMP	-234.17792	165.51905	2.00084	0.718	0.05	0	-724.24538	255.88954
446 D/E	wo	446 E/E	wo	-454.87816	143.62449	4.47901	0.02952	0.05	1	-880.12039	-29.63592
446 D/E	wo	446 E/E	8Br-cAMP	-371.84384	165.51905	3.17708	0.23516	0.05	0	-861.9113	118.22362
446 D/E	8Br-cAMP	446 D/D (WT)	wo	-281.71554	165.51905	2.40701	0.53685	0.05	0	-771.783	208.35192
446 D/E	8Br-cAMP	446 D/D (WT)	8Br-cAMP	-46.39229	165.51905	0.39638	0.99975	0.05	0	-536.45975	443.67517
446 D/E	8Br-cAMP	446 E/E	wo	-267.09253	143.62449	2.62995	0.43816	0.05	0	-692.33476	158.1497
446 D/E	8Br-cAMP	446 E/E	8Br-cAMP	-184.05821	165.51905	1.57261	0.87418	0.05	0	-674.12567	306.00925
446 D/E	8Br-cAMP	446 D/E	wo	187.78563	160.57706	1.65384	0.84911	0.05	0	-287.64964	663.2209

30 mV

Genotype	8Br-cAMP	Genotype	8Br-cAMP	MeanDiff	SEM	q Value	Prob	Alpha	Sig*	LCL	UCL
446 D/D (WT)	8Br-cAMP	446 D/D (WT)	wo	-611.28074	135.17107	6.39546	5.16769E-4	0.05	1	-1011.80142	-210.76005
446 E/E	wo	446 D/D (WT)	wo	-10.68861	117.29089	0.12888	1	0.05	0	-358.22915	336.85193
446 E/E	wo	446 D/D (WT)	8Br-cAMP	600.59213	121.78641	6.97422	1.31896E-4	0.05	1	239.73106	961.45319
446 E/E	8Br-cAMP	446 D/D (WT)	wo	-147.30945	140.18943	1.48604	0.89809	0.05	0	-562.69984	268.08095
446 E/E	8Br-cAMP	446 D/D (WT)	8Br-cAMP	463.97129	143.97171	4.55752	0.02569	0.05	1	37.37375	890.56883
446 E/E	8Br-cAMP	446 E/E	wo	-136.62084	127.33337	1.51736	0.88976	0.05	0	-513.9179	240.67623
446 D/E	wo	446 D/D (WT)	wo	-228.31056	131.1352	2.46219	0.51212	0.05	0	-616.8727	160.25157
446 D/E	wo	446 D/D (WT)	8Br-cAMP	382.97017	135.17107	4.00679	0.06846	0.05	0	-17.55051	783.49086
446 D/E	wo	446 E/E	wo	-217.62195	117.29089	2.62394	0.44095	0.05	0	-565.16249	129.91859
446 D/E	wo	446 E/E	8Br-cAMP	-81.00112	140.18943	0.81713	0.99206	0.05	0	-496.39151	334.38928
446 D/E	8Br-cAMP	446 D/D (WT)	wo	-590.27542	135.17107	6.1757	8.56646E-4	0.05	1	-990.7961	-189.75474
446 D/E	8Br-cAMP	446 D/D (WT)	8Br-cAMP	21.00532	139.08989	0.21357	0.99999	0.05	0	-391.12707	433.1377
446 D/E	8Br-cAMP	446 E/E	wo	-579.58681	121.78641	6.7303	2.3583E-4	0.05	1	-940.44788	-218.72574
446 D/E	8Br-cAMP	446 E/E	8Br-cAMP	-442.96597	143.97171	4.35119	0.03757	0.05	1	-869.56352	-16.36843
446 D/E	8Br-cAMP	446 D/E	wo	-361.96486	135.17107	3.78702	0.09792	0.05	0	-762.48554	38.55583

40 mV

Genotype	8Br-cAMP	Genotype	8Br-cAMP	MeanDiff	SEM	q Value	Prob	Alpha	Sig*	LCL	UCL
446 D/D (WT)	8Br-cAMP	446 D/D (WT)	wo	-1162.66956	154.96673	10.61043	8.87924E-8	0.05	1	-1622.99507	-702.34406
446 E/E	wo	446 D/D (WT)	wo	-167.59759	130.1981	1.82045	0.79038	0.05	0	-554.34838	219.15319
446 E/E	wo	446 D/D (WT)	8Br-cAMP	995.07197	145.5659	9.6674	2.86888E-7	0.05	1	562.67145	1427.47249
446 E/E	8Br-cAMP	446 D/D (WT)	wo	-524.01664	162.38556	4.56365	0.02605	0.05	1	-1006.37962	-41.65367
446 E/E	8Br-cAMP	446 D/D (WT)	8Br-cAMP	638.65292	174.94844	5.16262	0.00811	0.05	1	118.97217	1158.33367
446 E/E	8Br-cAMP	446 E/E	wo	-356.41905	153.43993	3.28502	0.20554	0.05	0	-812.20922	99.37112
446 D/E	wo	446 D/D (WT)	wo	-349.11278	140.63002	3.51077	0.1502	0.05	0	-766.85137	68.62581
446 D/E	wo	446 D/D (WT)	8Br-cAMP	813.55678	154.96673	7.42445	5.0885E-5	0.05	1	353.23128	1273.88229
446 D/E	wo	446 E/E	wo	-181.51519	130.1981	1.97162	0.73024	0.05	0	-568.26597	205.23559
446 D/E	wo	446 E/E	8Br-cAMP	174.90386	162.38556	1.52324	0.888	0.05	0	-307.45911	657.26684
446 D/E	8Br-cAMP	446 D/D (WT)	wo	-1010.96735	162.38556	8.8045	1.84059E-6	0.05	1	-1493.33032	-528.60438
446 D/E	8Br-cAMP	446 D/D (WT)	8Br-cAMP	151.70221	174.94844	1.2263	0.95245	0.05	0	-367.97854	671.38296
446 D/E	8Br-cAMP	446 E/E	wo	-843.36976	153.43993	7.77311	2.20856E-5	0.05	1	-1299.15993	-387.57959

446 D/E	8Br-cAMP	446 E/E	8Br-cAMP	-486.95071	181.55257	3.79313	0.09812	0.05	0	-1026.24891	52.3475
446 D/E	8Br-cAMP	446 D/E	wo	-661.85457	162.38556	5.76408	0.00229	0.05	1	-1144.21754	-179.49159

50 mV

Genotype	8Br-cAMP	Genotype	8Br-cAMP	MeanDiff	SEM	q Value	Prob	Alpha	Sig*	LCL	UCL
446 D/D (WT)	8Br-cAMP	446 D/D (WT)	wo	-1241.83854	161.39669	10.88142	7.69426E-8	0.05	1	-1721.2641	-762.41298
446 E/E	wo	446 D/D (WT)	wo	-171.80509	133.70374	1.81722	0.7916	0.05	0	-568.96931	225.35913
446 E/E	wo	446 D/D (WT)	8Br-cAMP	1070.03345	149.91182	10.09431	1.45558E-7	0.05	1	624.72346	1515.34343
446 E/E	8Br-cAMP	446 D/D (WT)	wo	-641.87332	161.39669	5.62432	0.0031	0.05	1	-1121.29888	-162.44777
446 E/E	8Br-cAMP	446 D/D (WT)	8Br-cAMP	599.96522	175.05929	4.84681	0.0152	0.05	1	79.95519	1119.97524
446 E/E	8Br-cAMP	446 E/E	wo	-470.06823	149.91182	4.43445	0.03303	0.05	1	-915.37822	-24.75825
446 D/E	wo	446 D/D (WT)	wo	-217.68347	146.46511	2.10187	0.67448	0.05	0	-652.75507	217.38814
446 D/E	wo	446 D/D (WT)	8Br-cAMP	1024.15507	161.39669	8.974	1.25274E-6	0.05	1	544.72951	1503.58063
446 D/E	wo	446 E/E	wo	-45.87838	133.70374	0.48527	0.99933	0.05	0	-443.0426	351.28584
446 D/E	wo	446 E/E	8Br-cAMP	424.18986	161.39669	3.7169	0.11046	0.05	0	-55.2357	903.61541
446 D/E	8Br-cAMP	446 D/D (WT)	wo	-819.52795	193.75513	5.98171	0.00142	0.05	1	-1395.07358	-243.98231
446 D/E	8Br-cAMP	446 D/D (WT)	8Br-cAMP	422.31059	205.27521	2.90945	0.32695	0.05	0	-187.45522	1032.0764
446 D/E	8Br-cAMP	446 E/E	wo	-647.72286	184.29789	4.97031	0.01193	0.05	1	-1195.17595	-100.26976
446 D/E	8Br-cAMP	446 E/E	8Br-cAMP	-177.65462	205.27521	1.22393	0.95283	0.05	0	-787.42043	432.11119
446 D/E	8Br-cAMP	446 D/E	wo	-601.84448	193.75513	4.39285	0.03561	0.05	1	-1177.39011	-26.29884

60 mV

Genotype	8Br-cAMP	Genotype	8Br-cAMP	MeanDiff	SEM	q Value	Prob	Alpha	Sig*	LCL	UCL
446 D/D (WT)	8Br-cAMP	446 D/D (WT)	wo	-1573.68844	214.2423	10.38792	1.25334E-7	0.05	1	-2215.55693	-931.81996
446 E/E	wo	446 D/D (WT)	wo	-178.32479	149.93838	1.68195	0.83922	0.05	0	-627.53916	270.88957
446 E/E	wo	446 D/D (WT)	8Br-cAMP	1395.36365	205.31158	9.61145	6.16391E-7	0.05	1	780.25155	2010.47575
446 E/E	8Br-cAMP	446 D/D (WT)	wo	-783.79138	198.34983	5.58835	0.004	0.05	1	-1378.04612	-189.53663
446 E/E	8Br-cAMP	446 D/D (WT)	8Br-cAMP	789.89707	242.92793	4.59841	0.02664	0.05	1	62.08661	1517.70752
446 E/E	8Br-cAMP	446 E/E	wo	-605.46658	188.66832	4.53844	0.02966	0.05	1	-1170.7156	-40.21757
446 D/E	wo	446 D/D (WT)	wo	-116.29995	166.38982	0.98848	0.98097	0.05	0	-614.80272	382.20282
446 D/E	wo	446 D/D (WT)	8Br-cAMP	1457.3885	217.6164	9.47106	8.34919E-7	0.05	1	805.41122	2109.36577
446 D/E	wo	446 E/E	wo	62.02484	154.72121	0.56693	0.99856	0.05	0	-401.51885	525.56854
446 D/E	wo	446 E/E	8Br-cAMP	667.49143	201.98958	4.67339	0.02327	0.05	1	62.33201	1272.65084
446 D/E	8Br-cAMP	446 D/D (WT)	wo	-752.85868	238.38682	4.46628	0.03369	0.05	1	-1467.064	-38.65336
446 D/E	8Br-cAMP	446 D/D (WT)	8Br-cAMP	820.82976	276.58562	4.197	0.05347	0.05	0	-7.81889	1649.47841
446 D/E	8Br-cAMP	446 E/E	wo	-574.53389	230.39392	3.52663	0.15092	0.05	0	-1264.79253	115.72474
446 D/E	8Br-cAMP	446 E/E	8Br-cAMP	30.93269	264.46644	0.16541	1	0.05	0	-761.40697	823.27235
446 D/E	8Br-cAMP	446 D/E	wo	-636.55874	241.42372	3.72884	0.11243	0.05	0	-1359.86258	86.74511

70 mV

Genotype	8Br-cAMP	Genotype	8Br-cAMP	MeanDiff	SEM	q Value	Prob	Alpha	Sig*	LCL	UCL
446 D/D (WT)	8Br-cAMP	446 D/D (WT)	wo	-2913.89038	423.71024	9.72566	1.73683E-6	0.05	1	-4202.64603	-1625.13472
446 E/E	wo	446 D/D (WT)	wo	-356.23303	174.30451	2.89028	0.34279	0.05	0	-886.39705	173.93099
446 E/E	wo	446 D/D (WT)	8Br-cAMP	2557.65735	417.14069	8.67111	1.34167E-5	0.05	1	1288.88361	3826.43108
446 E/E	8Br-cAMP	446 D/D (WT)	wo	-770.26083	267.97789	4.06494	0.0725	0.05	0	-1585.34148	44.81981
446 E/E	8Br-cAMP	446 D/D (WT)	8Br-cAMP	2143.62954	464.15131	6.53138	8.86938E-4	0.05	1	731.86846	3555.39063
446 E/E	8Br-cAMP	446 E/E	wo	-414.0278	257.46482	2.27419	0.5998	0.05	0	-1197.13195	369.07635
446 D/E	wo	446 D/D (WT)	wo	-298.06819	189.48898	2.22457	0.62172	0.05	0	-874.41724	278.28086
446 D/E	wo	446 D/D (WT)	8Br-cAMP	2615.82219	423.71024	8.7308	1.19388E-5	0.05	1	1327.06653	3904.57784
446 D/E	wo	446 E/E	wo	58.16484	174.30451	0.47192	0.99939	0.05	0	-471.99918	588.32886
446 D/E	wo	446 E/E	8Br-cAMP	472.19264	267.97789	2.49193	0.50397	0.05	0	-342.888	1287.27328
446 D/E	8Br-cAMP	446 D/D (WT)	wo	-2913.89038	423.71024	9.72566	1.73683E-6	0.05	1	-4202.64603	-1625.13472
446 D/E	8Br-cAMP	446 D/D (WT)	8Br-cAMP	0	568.46694	0	1	0.05	0	-1729.04715	1729.04715
446 D/E	8Br-cAMP	446 E/E	wo	-2557.65735	417.14069	8.67111	1.34167E-5	0.05	1	-3826.43108	-1288.88361
446 D/E	8Br-cAMP	446 E/E	8Br-cAMP	-2143.62954	464.15131	6.53138	8.86938E-4	0.05	1	-3555.39063	-731.86846
446 D/E	8Br-cAMP	446 D/E	wo	-2615.82219	423.71024	8.7308	1.19388E-5	0.05	1	-3904.57784	-1327.06653

*1 indicates that the difference of the means is significant (P<0.05); 0 indicates that the difference of the means is not significant (P>0.05). wo: without 8Br-cAMP; LCL: Lower confidence level; UCL: Upper confidence level.

Table S2. *P* values for $V_{1/2}$ comparisons among genotypes using Tukey´s comparison test from two-way ANOVA with and without 8Br-cAMP.

Genotype	cAMP	Genotype	cAMP		MeanDiff	SEM	q Value	Prob	Alpha	Sig*	LCL	UCL
446 D/D (WT)	cAMP	446 D/D (WT)	wo		-36.1713	3.69877	13.82998	3.56E-08	0.05	1	-47.0227	-25.3198
446 E/E	wo	446 D/D (WT)	wo		-14.728	3.12001	6.67578	1.75E-04	0.05	1	-23.8815	-5.57446
446 E/E	wo	446 D/D (WT)	cAMP		21.44327	4.07123	7.4487	2.29E-05	0.05	1	9.49906	33.38748
446 E/E	cAMP	446 D/D (WT)	wo		-22.1816	3.89188	8.06026	4.33E-06	0.05	1	-33.5996	-10.7636
446 E/E	cAMP	446 D/D (WT)	cAMP		13.98964	4.68904	4.21927	0.044	0.05	1	0.23288	27.7464
446 E/E	cAMP	446 E/E	wo		-7.45363	4.24744	2.48174	0.50138	0.05	0	-19.9148	5.00755
446 D/E	wo	446 D/D (WT)	wo		-1.32458	3.20323	0.5848	0.99838	0.05	0	-10.7222	8.07307
446 D/E	wo	446 D/D (WT)	cAMP		34.84667	4.13535	11.91692	2.13E-08	0.05	1	22.71434	46.97899
446 D/E	wo	446 E/E	wo		13.4034	3.62694	5.22624	0.0057	0.05	1	2.76265	24.04415
446 D/E	wo	446 E/E	cAMP		20.85702	4.30893	6.84538	1.13E-04	0.05	1	8.21543	33.49862
446 D/E	cAMP	446 D/D (WT)	wo		-35.0154	3.5413	13.98336	3.51E-08	0.05	1	-45.4049	-24.6259
446 D/E	cAMP	446 D/D (WT)	cAMP		1.15583	4.40241	0.3713	0.99982	0.05	0	-11.76	14.07168
446 D/E	cAMP	446 E/E	wo		-20.2874	3.92872	7.30283	3.39E-05	0.05	1	-31.8136	-8.76132
446 D/E	cAMP	446 E/E	cAMP		-12.8338	4.56586	3.9751	0.06799	0.05	0	-26.2292	0.56155
446 D/E	cAMP	446 D/E	wo		-33.6908	3.99513	11.92604	2.12E-08	0.05	1	-45.4118	-21.9699

*1 indicates that the difference of the means is significant (P<0.05); 0 indicates that the difference of the means is not significant (P>0.05). wo: without 8Br-cAMP; LCL: Lower confidence level; UCL: Upper confidence level.

Table S3. Clinical and ECG characteristics of both index cases and available family members.

Individual	Age	*KCNQ1* Genotype	SCD	Prolonged QT interval	Early Repolarization V1-V4	Sinus Bradycardia	Other studies
Index case	18	p.D446E /WT	Yes	Yes	Yes	-	EP: QTc 520ms
II-2	42	p.D446E /WT	No	No	Yes	Yes	-
I-2	71	p.D446E /WT	No	Yes	No	Yes	-
II-6	32	p.D446E /WT	No	No	No	No	-
III-1	26	p.D446E /WT	No	No	No	No	-
II-4	40	WT/WT	No	No	No	No	-
III-3	11	WT/WT	No	No	No	No	-
III-4	4	WT/WT	No	No	Yes	Yes	-
III-5	8	WT/WT	No	No	Yes	No	-
IV-1	5	WT/WT	No	No	Yes	No	-
IV-2	3	WT/WT	No	No	Yes	No	-

Family 2

Index case	8	p.D446E /WT	No	No	No	No	Viskin: QTc 478ms
I-1	37	p.D446E /WT	No	No	Yes	No	No
